# Signal Peptide Peptidase and PI4Kβ1/2 play opposite roles in plant ER stress response and immunity

**DOI:** 10.1007/s44154-024-00155-z

**Published:** 2024-03-20

**Authors:** Karen Thulasi Devendrakumar, Tony ShengZhe Peng, Leon Pierdzig, Edan Jackson, Volker Lipka, Xin Li

**Affiliations:** 1 Michael Smith Laboratories, University of British Columbia, Vancouver, BC V6T 1Z4 Canada; 2 Department of Botany, University of British Columbia, Vancouver, BC V6T 1Z4 Canada; 3 Department of Plant Cell Biology, Georg August Universität Göttingen, 37077 Göttingen, Lower Saxony Germany

**Keywords:** Signal peptide peptidase, Plant immunity, *pi4kβ1,2*, *PI4Kβ*, ER stress, EMS mutagenesis

## Abstract

The Arabidopsis *pi4kβ1,2* mutant is mutated in the phosphatidylinositol 4-kinase (PI4K) β1 and PI4Kβ2 enzymes which are involved in the biosynthesis of phosphatidylinositol 4-phosphate (PI4P), a minor membrane lipid with important signaling roles. *pi4kβ1,2* plants display autoimmunity and shorter roots. Though the *pi4kβ1,2* mutant has been extensively characterized, the source of its autoimmunity remains largely unknown. In this study, through a genetic suppressor screen, we identified multiple partial loss-of-function alleles of *signal peptide peptidase* (*spp*) that can suppress all the defects of *pi4kβ1,2*. SPP is an intramembrane cleaving aspartic protease. Interestingly, *pi4kβ1,2* plants display enhanced ER stress response and mutations in *SPP* can suppress such phenotype. Furthermore, reduced ER stress responses were observed in the *spp* single mutants. Overall, our study reveals a previously unknown function of PI4Kβ and SPP in ER stress and plant immunity.

**Supplementary Information:**

The online version contains supplementary material available at 10.1007/s44154-024-00155-z.

## Introduction

Plant immunity is complex and involves proteins with roles in pathogen detection and defense activation (Jones & Dangl 2006). The recognition of molecules derived from pathogens/microbes results in elicitation of Pattern Triggered Immunity (PTI) (Monaghan & Zipfel 2012; Bigeard et al. 2015). Successful pathogens can deliver effectors into plant cells to dampen PTI. Perception of these effector molecules by plants results in Effector Triggered Immunity (ETI) (van Wersch et al. 2020; Zhou & Zhang 2020). Further, PTI and ETI are intricately connected and have been shown to mutually potentiate each other (Ngou et al. 2021; Pruitt et al. 2021; Tian et al. 2021; Yuan et al. 2021b,a).

Immune responses need to be tightly controlled since any dysregulation can lead to either an insufficient or an excessive immune output. Autoimmune mutants often display a smaller size proportional to the level of constitutive immunity, making them a valuable resource for genetic analyses. They have been successfully used in genetic screens to identify contributors mediating immunity (Johnson et al. 2012; van Wersch et al. 2016).

One such autoimmune mutant is *pi4kβ1,2* (Janda et al. 2014; Šašek et al. 2014; Kalachova et al. 2020). *pi4kβ1,2* is mutated in the redundant phosphatidylinositol 4-phosphate (PI4P) biosynthetic enzymes, Phosphatidylinositol 4-Kinase (PI4K) β1 and PI4Kβ2. *pi4kβ1,2* plants display autoimmunity-associated dwarfism and enhanced resistance against bacterial and oomycete pathogens (Janda et al. 2014; Šašek et al. 2014; Antignani et al. 2015; Thulasi Devendrakumar et al. 2023). In addition to displaying autoimmunity and dwarfism, *pi4kβ1,2* seedlings also grow shorter roots with abnormal root hairs (Preuss et al. 2006; Šašek et al. 2014; Starodubtseva et al. 2022; Thulasi Devendrakumar et al. 2023). Through reverse genetic analysis it has been shown that the defense hormone salicylic acid (SA) and *Enhanced Disease Susceptibility 1* (*EDS1*), a central component required for ETI, are both contributing to *pi4kβ1,2*’s autoimmunity (Šašek et al. 2014). However, the exact cause of the autoimmunity displayed by *pi4kβ1,2* is still unclear (Šašek et al. 2014). Additionally, no genes required for *pi4kβ1,2*’s short root defect have been identified so far. Thus, we conducted an EMS forward genetic screen aiming to identify novel genes involved in *pi4kβ1,2*’s autoimmunity (Thulasi Devendrakumar et al. 2024).

Here, we report that Signal Peptide Peptidase (SPP) is required for *pi4kβ1,2* autoimmunity and root length defect. SPP is annotated as an intramembrane cleaving aspartic protease (Ponting et al. 2002; Weihofen et al. 2002; Tamura et al. 2008). Arabidopsis SPP has been shown to be ER localized and an essential gene required for the formation of viable pollen (Tamura et al. 2008; Han et al. 2009). Since knockout of SPP leads to lethality, functional characterization of Arabidopsis SPP is lacking. From our *pi4kβ1,2* suppressor screen, we identified three *spp* partial loss-of-function alleles that suppress all the *pi4kβ1,2* defects. Further, we uncovered that both PI4Kβ1,2 and SPP contribute to ER stress responses.

## Results

### Identification of suppressors 79-1, 145-1, and 171-1 from the *pi4kβ1,2* suppressor screen

**Fig. 1:**
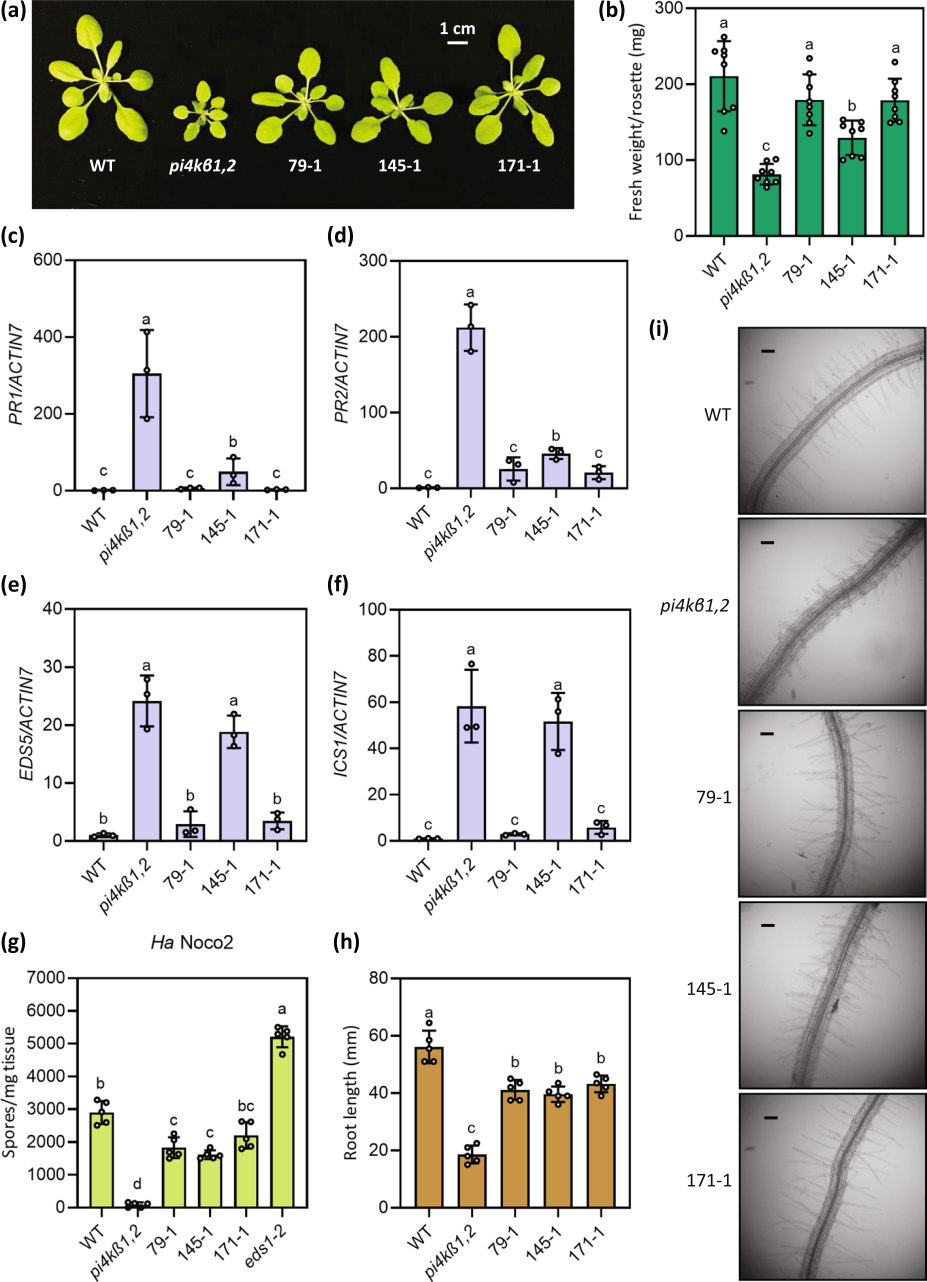
Characterization of suppressors of *pi4kβ1,2*. **a** Morphology of 4-week-old WT, *pi4kβ1,2*, 79–1, 145–1, and 171–1 plants. **b** Quantification of rosette weight of 4-week-old plants of the indicated genotypes. The error bars represent SD of the replicates (*n* = 8). **c **Pathogenesis Related 1 ( PR1 ) expression in plants of the indicated genotypes. PR1 expression was normalised to the expression level of ACTIN7 . The error bars represent SD of the biological replicates (*n* = 3). **d** Pathogenesis Related 2 ( PR2 ) expression in plants of the indicated genotypes. PR2 expression was normalised to the expression level of ACTIN7. The error bars represent SD of the biological replicates (*n* = 3). **e **Enhanced Disease Susceptibility 5 ( EDS5 ) expression in plants of the indicated genotypes. EDS5 expression was normalised to the expression level of ACTIN7 . The error bars represent SD of the biological replicates (*n* = 3). **f **Isochorismate Synthase 1 ( ICS1 ) expression in plants of the indicated genotypes. ICS1 expression was normalised to the expression level of ACTIN7 . The error bars represent SD of the biological replicates (*n* = 3). **g** Quantification of *Hyaloperonospora arabidopsidis* (*Ha*) Noco2 growth on plants of the indicated genotypes. The error bars represent SD of the biological replicates (*n* = 5). **h** Root lengths of 10-day-old plate-grown seedlings of the indicated genotypes. The error bars represent SD of the replicates (*n* = 5). From (**b** to **h**), the letters indicate significant difference between the different genotypes as determined using a one-way ANOVA with post-hoc Tukey’s Honestly Significant Difference (HSD) test. Genotypes denoted with the different letters have significant difference (*p *<0.05). **i** Bright field microscopy images showing root hair morphology of plate grown 14-day-old seedlings of the indicated genotypes. The scale bar indicates 100 μm

An ethyl methane sulfonate (EMS) mutagenesis based forward genetic screen was carried out to investigate the source of *pi4kβ1,2* autoimmunity (Thulasi Devendrakumar et al. 2024). Suppressors 79-1, 145–1, and 171–1 were identified (Fig. 1a). 79-1 and 171-1, and to a lesser degree 145-1 suppressed the autoimmunity-associated dwarfism of *pi4kβ1,2* (Fig. 1a, b). Further, they displayed reduced expression of the defense marker gene *Pathogenesis related 1* (*PR1*), and *PR2* when compared to *pi4kβ1,*2 (Fig. 1c, d). *pi4kβ1,2* was also reported to accumulate salicylic acid (SA), with increased transcript levels of the SA biosynthetic gene *Isochorismate synthase 1* (*ICS1*) (Wildermuth et al. 2002; Janda et al. 2014; Šašek et al. 2014). The suppressors 79-1 and 171-1 displayed reduced expression of *ICS1* and *Enhanced Disease Susceptibility 5* (*EDS5*), a gene that encodes a protein required for the transport of the SA precursor isochorismate from the chloroplast to the cytoplasm (Fig. 1e, f; Rekhter et al. 2019). Consistently, the suppressors also supported more growth of the oomycete pathogen *Hyaloperonospora arabidopsidis* Noco2 (*Ha* Noco2) compared to *pi4kβ1,*2 (Fig. 1g). In addition to suppression of autoimmunity and the associated dwarfism, these suppressors displayed suppression of *pi4kβ1,2*’s short root phenotype (Fig. 1h). Further, they showed suppression of the root hair defects of *pi4kβ1,2* (Fig. 1i). Thus suppressors 79–1, 145–1, and 171–1 show suppression of both the autoimmunity and root defects of *pi4kβ1,2*.

### Suppressor mutations identified by mapping-by-sequencing

To identify the causal mutations that led to the suppression of *pi4kβ1,2* phenotypes, we performed mapping-by-sequencing. The suppressor mutations present in 79–1, 145–1, and 171–1 will hence be referred to as *79–1*, *145–1*, and *171–1*. The suppressors were backcrossed to *pi4kβ1,2* to establish their dominance, to determine whether the suppression is caused by a single nuclear mutation, and to generate mapping populations for whole genome sequencing.

F1 plants generated by crossing either 79–1 or 145–1 to *pi4kβ1,2* appeared *pi4kβ1,*2-like, suggesting that the suppressor mutations are recessive. In F2, the 79–1 × *pi4kβ1,2* F2 population showed a 127:41 segregation of *pi4kβ1,2*-like: suppressor-like plants, consistent with the expected 3:1 ratio for recessive mutations (χ2 = 0.032; *P* value = 0.8586). The 145–1 × *pi4kβ1,2* F2 population showed a 109:36 segregation of *pi4kβ1,2*-like: suppressor-like plants that was also consistent with the expected 3:1 ratio for recessive mutations (χ2 = 0.002; *P* value = 0.9618). Thus, 79–1 and 145–1 carry single nuclear recessive mutations. For each mutant, tissue was collected from the suppressor-like F2 plants for DNA extraction and whole genome sequencing.

The F1 generated by backcrossing 171–1 to *pi4kβ1,2* appeared suppressor-like, indicating that it is dominant. Further, in the 171–1 × *pi4kβ1,2* F2 population we observed a 53:151 segregation of *pi4kβ1,2*-like: suppressor-like plants, consistent with the expected 1:3 ratio for a single dominant nuclear mutation (χ2 = 0.105; *P* value = 0.7464). F3 seeds from the suppressor-like F2 plants were harvested and planted. Of the 151 F3 populations, 43 displayed no segregation of plants with *pi4kβ1,2*-like phenotype, demonstrating homozygosity of the suppressor locus. Tissue was collected from these 43 homozygous F3 populations for DNA extraction and whole genome sequencing.

**Fig. 2:**
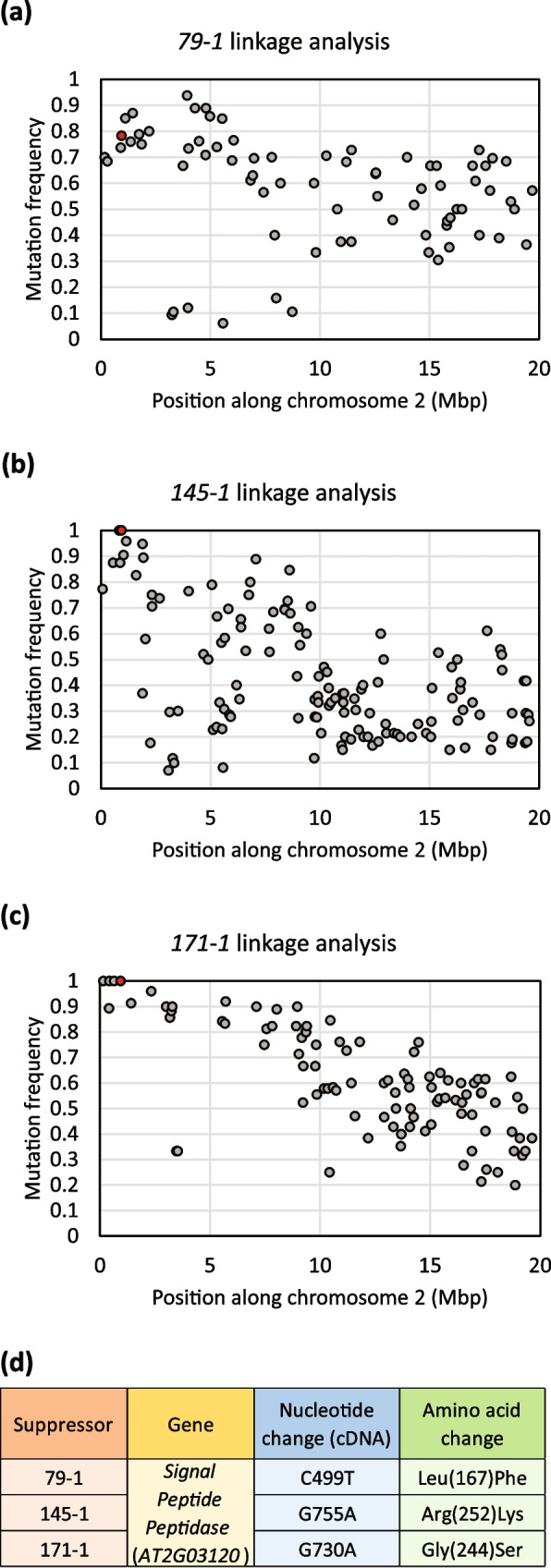
Mapping by next-generation-sequencing of suppressors 79–1, 145–1, and 171–1. **a**, **b**, **c** Linkage map showing the linkage region for the suppressors 79–1 (**a**), 145–1 (**b**), and 171–1 (**c**) to the beginning of chromosome 2. The *Signal Peptide Peptidase* (*SPP*) mutation is highlighted using a red data point. **d ***SPP* mutations in the suppressors and their resulting amino acid changes

A shared linkage region was identified for these three suppressors on chromosome 2 (Fig. 2a, b, c). Further, they carried different mutations in *Signal Peptide Peptidase* (*SPP*; *AT2G03120*; Fig. 2d) within the identified linkage regions. These mutations all resulted in single amino acid substitutions in SPP (Fig. 2d).

### Mutations in *Signal Peptide Peptidase* are the causal mutations responsible for the *pi4kβ1,2* suppression in 79–1, 145–1 and 171–1

**Fig. 3:**
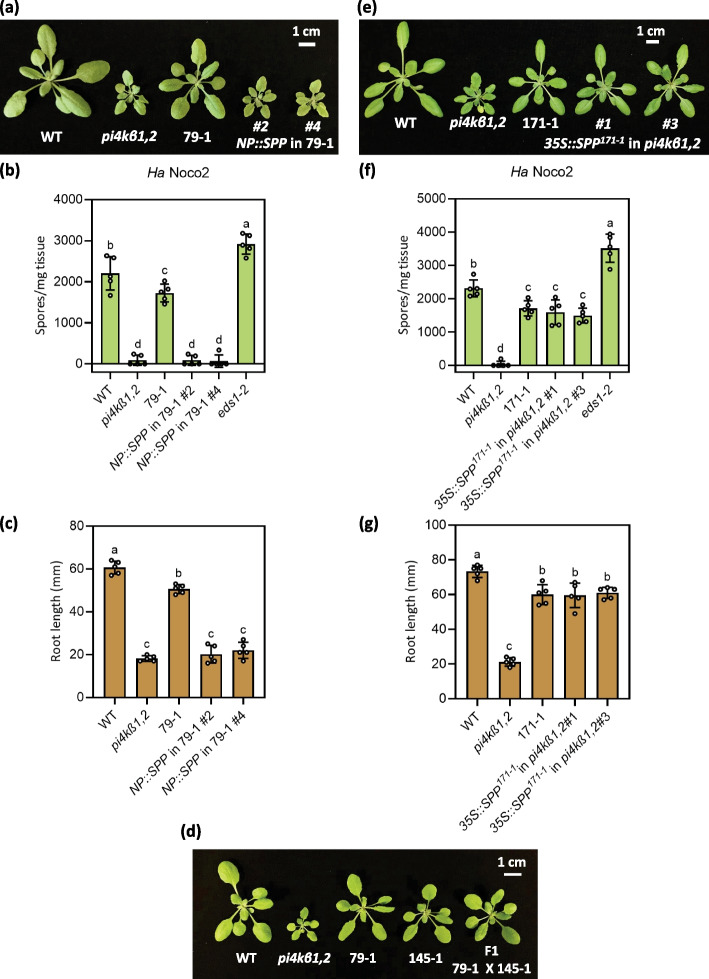
Mutations in *SPP* are responsible for the suppression phenotypes seen in 79–1, 145–1, and 171–1. **a** Morphology of 4-week-old WT, *pi4kβ1,2*, 79–1, and two independent *NP::SPP* transgenic lines in the 79–1 background. **b** Quantification of *Ha* Noco2 growth on plants of the indicated genotypes. The error bars represent SD of the biological replicates (*n* = 5). **c** Root lengths of 10-day-old plate-grown seedlings of the indicated genotypes. The error bars represent SD of the replicates (*n* = 5). **d** Morphology of 4-week-old WT, *pi4kβ1,2*, 79–1, 145–1, and 79–1 × 145–1 F1 plants. **e** Morphology of 4-week-old WT, *pi4kβ1,2*, 171–1, and two independent *35S::SPP*^*171−1*^ transgenic lines in the *pi4kβ1,2* background. **f** Quantification of *Ha* Noco2 growth on plants of the indicated genotypes. The error bars represent SD of the biological replicates (*n* = 5). **g** Root lengths of 10-day-old plate-grown seedlings of the indicated genotypes. The error bars represent SD of the replicates (*n* = 5). In (**a** to **c**) and (**e** to **g**), the letters indicate significant difference between the different genotypes as determined using a one-way ANOVA with post-hoc Tukey’s Honestly Significant Difference (HSD) test. Genotypes denoted with the different letters have significant difference (*p* < 0.05)

To confirm that the mutations in *SPP* are causing the *pi4kβ1,2* suppression phenotypes, we transformed the 79–1 and 171–1 plants with *NP::SPP* driven by its native promoter (NP). These *NP::SPP* transgenic plants displayed a *pi4kβ1,2*-like size (Fig. 3a, S1a). Further, the autoimmunity and the root length returned to *pi4kβ1,2* levels in the transgenic lines (Fig. 3b, c, S1b, c). When the recessive suppressors 79–1 and 145–1 were crossed, the F1 progeny appeared suppressor like (Fig. 3d), further confirming that 79–1 and 145–1 carry mutant alleles of the same gene. In addition, to test that the *SPP* mutation in 171–1 is the dominant suppressor mutation responsible for the suppression we transformed *pi4kβ1,2* with *35S::SPP*^*171−1*^*,* cloned from 171–1 genomic DNA. The resulting transgenic plants displayed suppression of *pi4kβ1,2* autoimmunity-associated dwarfism, immunity, and root length defect (Fig. 3e-g). These results confirmed that the *SPP* mutation in 171–1 is the dominant mutation responsible for the suppression of *pi4kβ1,2* phenotypes. Further, since the suppressor 171–1 resembles the recessive alleles 79–1, and 145–1, it likely carries a dominant-negative (DN) allele of *SPP*. Taken together, the mutations in *SPP* are responsible for the *pi4kβ1,2* suppression seen in 79–1, 145-1, and 171–1. We named the *spp* alleles *79–1*, *145–1*, and *171–1* as *spp-3*, *spp-4*, and *spp-5* respectively. As knockout (KO) of this gene is lethal (Tamura et al. 2008; Han et al. 2009), these alleles are likely partial loss-of-function alleles of *SPP*.

### SPP and PI4Kβ1 are localised to the ER

**Fig. 4:**
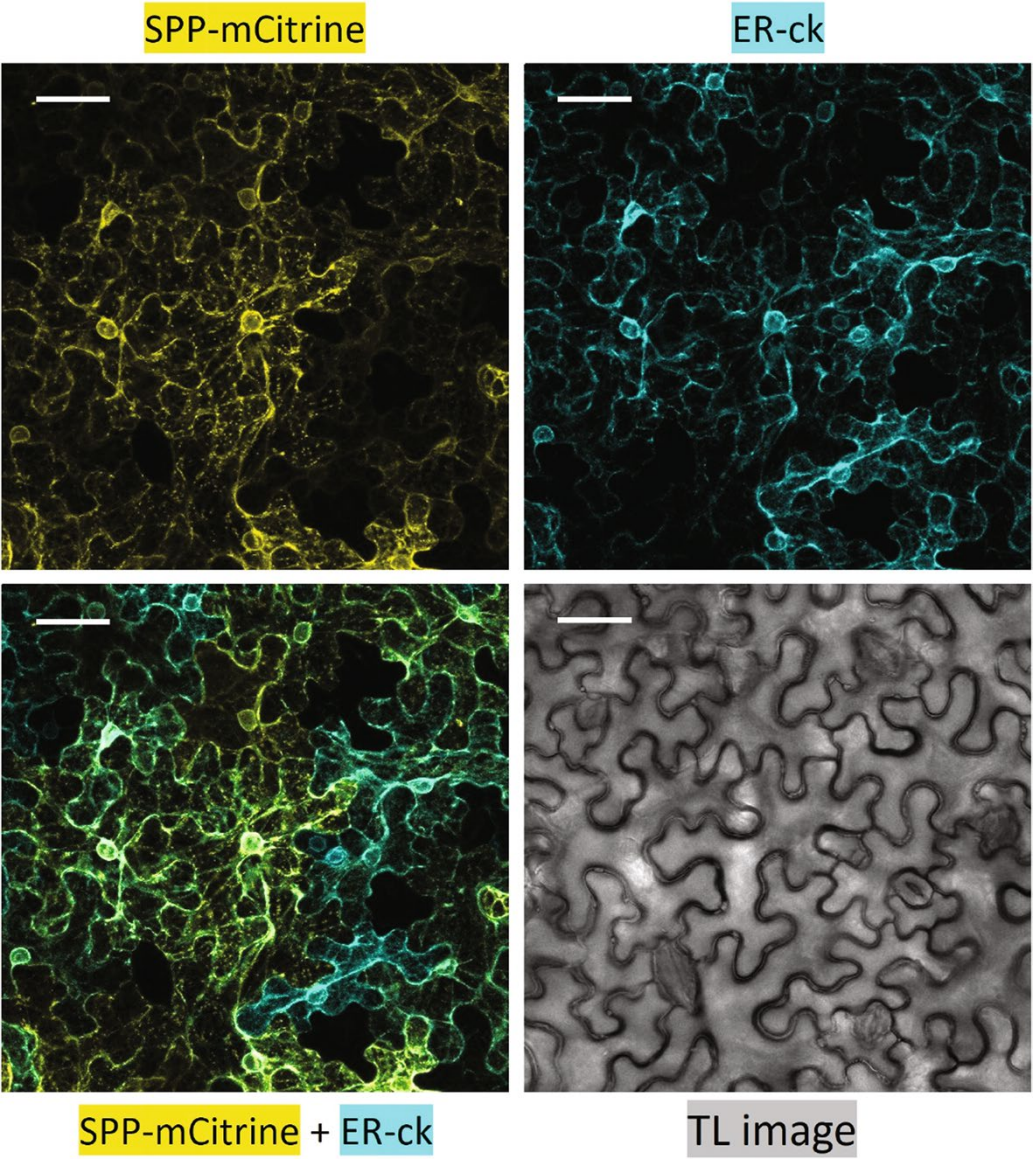
SPP is localized to the ER. Co-localization of SPP-mCitrine with the endoplasmic reticulum (ER) marker ER-ck following transient expression in *Nicotiana benthamiana* leaf epidermal cells. Clockwise from top-left, images show SPP-mCitrine fluorescence in yellow, ER-ck fluorescence in cyan, the transmission light (TL) image in greyscale, and overlay of mCitrine and CFP channels. The colocalization of yellow and cyan signals appear as green. The confocal images are 25 μm Z-stacks while the TL image is of a single plane. The scale bars are 40 μm

**Fig. 5:**
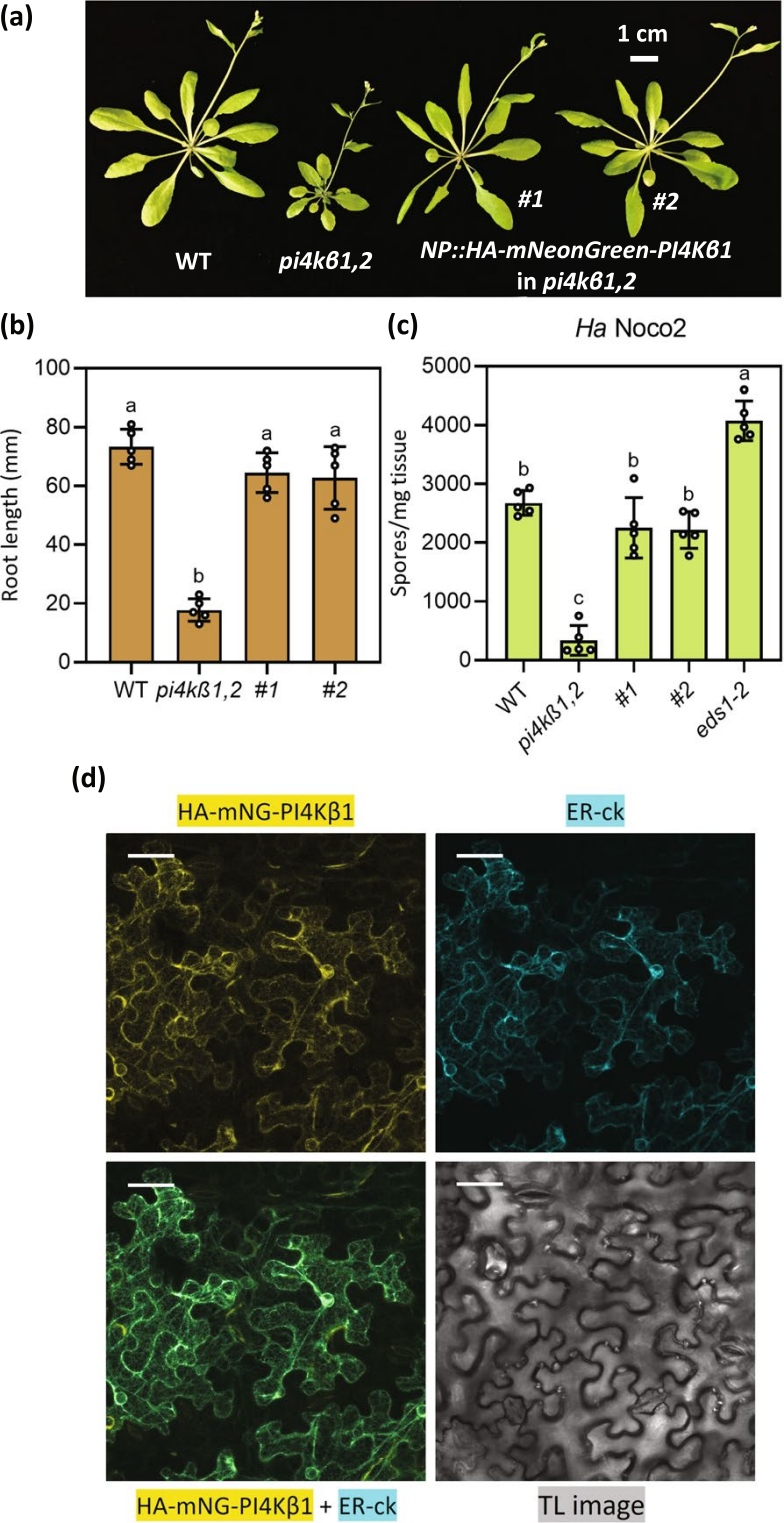
PI4Kβ1 is localized to the ER. **a** Morphology of 5-week-old WT, *pi4kβ1,2*, and two independent *NP::HA-mNeonGreen-PI4Kβ1* transgenic lines in the *pi4kβ1,2* background. **b** Root lengths of 10-day-old plate-grown seedlings of the indicated genotypes. The error bars represent SD of the replicates (*n* = 5). In (**b**-**c)** the letters indicate significant difference between the different genotypes as determined using a one-way ANOVA with post-hoc Tukey’s Honestly Significant Difference (HSD) test. Genotypes denoted with the different letters have significant difference (*p* < 0.05). **c** Quantification of *Ha* Noco2 growth on plants of the indicated genotypes. The error bars represent SD of the biological replicates (*n* = 5). **d** Co-localization of HA-mNG-PI4Kβ1 with the endoplasmic reticulum (ER) marker ER-ck following transient expression in *N. benthamiana* leaf epidermal cells. Clockwise from top-left, images show HA-mNG-PI4Kβ1 fluorescence in yellow, ER-ck fluorescence in cyan, the transmission light (TL) image in greyscale, and overlay of mNG and CFP channels. The colocalization of yellow and cyan signals appear as green. The confocal images are 25 μm Z-stacks while the TL image is of a single plane. The scale bars are 40 μm

SPP is reported to be an intramembrane cleaving aspartyl protease and was previously reported to be ER localised in Arabidopsis (Tamura et al. 2008). In order to verify its localization, we generated an SPP-mCitrine fusion. We transiently co-expressed SPP-mCitrine (Yellow; Fig. 4) and the ER marker ER-ck (Cyan; Fig. 4; Nelson et al. 2007) in *Nicotiana benthamiana* leaves to test whether the two proteins colocalize. As shown in Fig. 4, a network-like localization of the SPP-mCitrine fusion similar to the ER marker ER-ck was observed in confocal microscopy studies (Fig. 4). Colocalization of the SPP-mCitrine and the ER-ck signal was also obvious (Fig. 4). Interestingly, in addition to signals clearly confined to the ER, SPP-mCitrine also displayed punctate localization that did not overlap with the signal of the ER marker (Fig. 4). We further investigated the localization of the PI4Kβs. PI4Kβ1 was previously shown to localize to distinct compartments of the trans Golgi network in Arabidopsis root hair cells (Kang et al. 2011; Antignani et al. 2015). We generated a native promoter driven *NP::HA-mNeonGreen*(*mNG*)*-PI4Kβ1* construct. The transgene was confirmed to be functional since it could complement the morphological, immune, and root phenotypes of *pi4kβ1,2* (Fig. 5a-c). Further, to check the localization of PI4Kβ1 we transiently expressed HA-mNG-PI4Kβ1 in *N. benthamiana* leaves. We observed a network like localization of PI4Kβ1 (Yellow; Fig. 5d). The signals of the HA-mNG-PI4Kβ1 and ER-ck largely overlapped, indicating that the PI4Kβ1 protein is localized to the ER (Fig. 5d). Thus, both SPP and PI4Kβ1 localize to the ER when transiently expressed in *N. benthamiana* leaves.

### Identified *SPP* mutations result in non-synonymous mutations of highly conserved residues

**Fig. 6:**
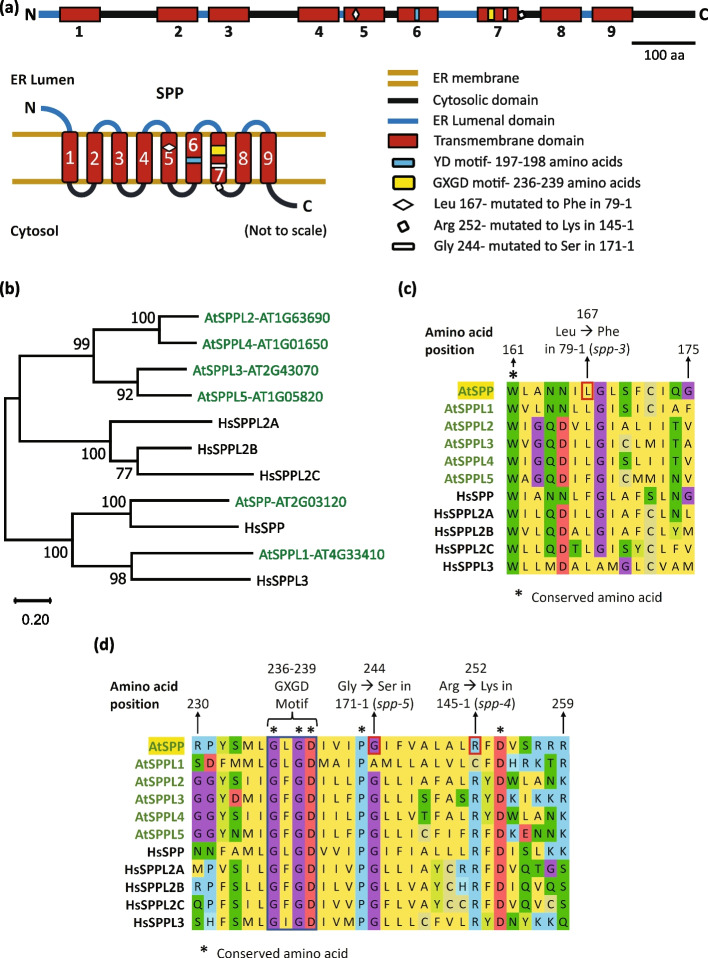
*spp-3*, *spp-4*, and *spp-5* mutations lead to missense mutations of conserved amino acids in SPP. **a** Diagram of SPP domain architecture with labeled active site motifs and the mutations sites. **b** Phylogeny of Arabidopsis and human SPP and SPPLs. The tree was constructed with full length amino acid sequences. The numbers above the branches indicate the bootstrap values from 1000 bootstrap replicates. The scale denotes branch length. The following abbreviations have been used in the tree to denote the organisms: At- *Arabidopsis thaliana* and Hs- *Homo sapiens*. **c** Multiple sequence alignment showing the region containing the *spp-3* mutation present in the suppressor 79–1. **d** Multiple sequence alignment showing the region containing the *spp-4* and *spp-5* mutations present in the suppressors 145–1 and 171–1 respectively

SPP is a multi-pass membrane protein with 9 transmembrane helices (Fig. 6a; Ponting et al. 2002; Weihofen et al. 2002; Tamura et al. 2008)*.* It is a presenilin type protease with two active aspartic acid residues present within the catalytic YD and the GXGD motifs present on its sixth and seventh transmembrane helices respectively (Fig. 6a; Ponting et al. 2002; Weihofen et al. 2002; Spasic et al. 2006). Arabidopsis SPP is an essential gene since the null *spp* alleles are lethal (Tamura et al. 2008; Han et al. 2009). To understand the consequence of the *spp* mutations identified in our screen on SPP function, we generated an amino acid sequence alignment and phylogenetic tree of Arabidopsis and human SPP and SPP-like (SPPL) proteins (Fig. 6b). The Arabidopsis SPP is most closely related to the human SPP (Fig. 6b). The Arabidopsis SPP homolog, SPP-Like (SPPL) 1 shows the highest homology to the Human SPPL3 (Fig. 6b). The other Arabidopsis SPP homologs, SPPL2-5 form a sister clade along with the human SPPL2a, 2b, and 2c (Fig. 6b). The mutation in *spp-3* allele leads to the Leu(167)Phe mutation of a fairly conserved amino acid present in the fifth transmembrane domain of SPP (Fig. 6c). Further, *spp-4* and *spp-5* mutations lead to missense mutations of highly conserved residues in the proximity of the GXGD active site in the seventh transmembrane domain (Fig. 6d). The amino acids mutated in the three *spp* alleles are largely conserved in all Arabidopsis and human SPP homologs, indicating that they are likely important for the function of SPP and SPPLs. Since complete loss of SPP function leads to lethality, the *spp* alleles likely have reduced SPP function due to mutations of amino acids that are in close proximity to its active sites.

### *SPP* transcription is up-regulated in *pi4kβ1,2*

**Fig. 7:**
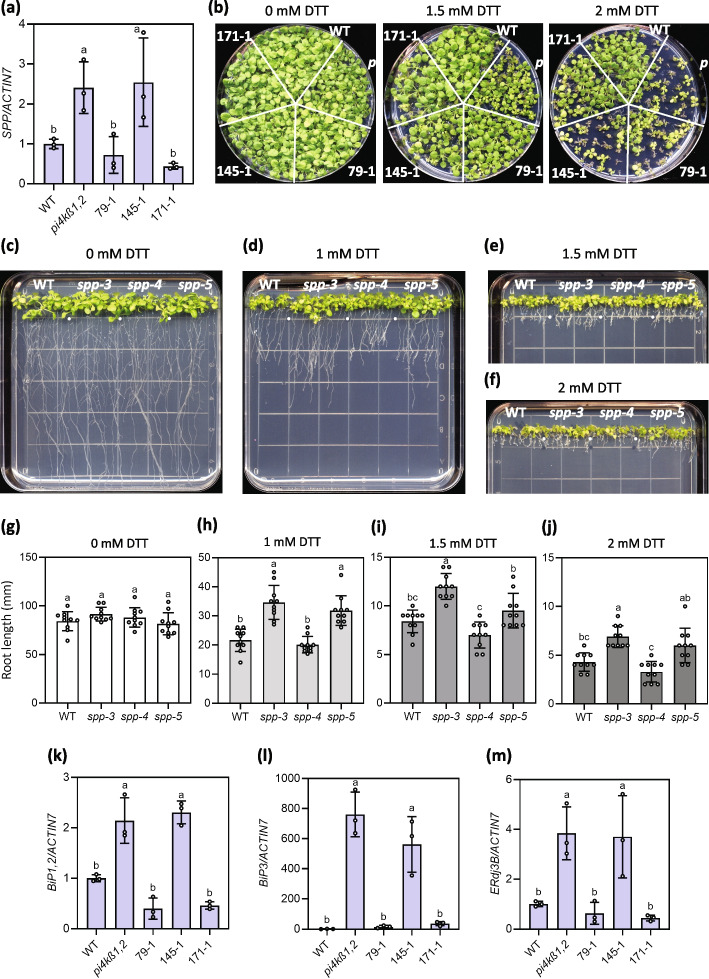
*SPP* mutations reduce ER stress sensitivity in both WT and *pi4kβ1,2. ***a** Expression of *SPP* in WT, *pi4kβ1,2*, 79–1, 145–1, and 171–1 normalised to the expression level of *ACTIN7*. The error bars represent SD of the biological replicates (*n* = 3). The letters indicate significant difference between the different genotypes as determined using a one-way ANOVA with post-hoc Tukey’s Honestly Significant Difference (HSD) test. Genotypes denoted with the different letters have significant difference (*p* < 0.05). **b**, 21-day-old WT, *pi4kβ1,2*, 79–1, 145–1, and 171–1 seedlings grown on ½ MS agar plates supplemented with the indicated concentration of DTT. **c,** **d**, **e**, **f**, 14-day-old WT, *spp-3*, *spp-4*, and *spp-5* seedlings grown vertically on square ½ MS agar plates supplemented with 0, 1, 1.5, and 2 mM DTT respectively. **g, h**, **i**, **j**, Quantification of the root lengths of the 14-day-old vertical plate grown WT, *spp-3*, *spp-4*, and *spp-5* seedlings shown in **c**-**f**. The error bars represent SD of the replicates (*n* = 10). The letters indicate significant difference between the different genotypes as determined using a one-way ANOVA with post hoc Tukey’s Honestly Significant Difference (HSD) test. Genotypes denoted with the different letters have significant difference (*p* < 0.01). **k** Expression of *BiP1,2* in WT, *pi4kβ1,2*, 79–1, 145–1, and 171–1 normalised to the expression level of *ACTIN7*. The error bars represent SD of the biological replicates (*n* = 3). **l** Expression of *BiP3* in WT, *pi4kβ1,2*, 79–1, 145–1, and 171–1 normalised to the expression level of *ACTIN7*. The error bars represent SD of the biological replicates (*n* = 3). **m** Expression of *ERdj3B* in WT, *pi4kβ1,2*, 79–1, 145–1, and 171–1 normalised to the expression level of *ACTIN7*. The error bars represent SD of the biological replicates (*n* = 3). In **k-m** genotypes denoted with the different letters have significant difference (*p* < 0.05) as determined using a one-way ANOVA with post-hoc Tukey’s Honestly Significant Difference (HSD) test

SPP has been shown to be involved in the unfolded protein response (UPR) and endoplasmic reticulum–associated protein degradation (ERAD) in humans, the human parasite *Plasmodium falciparum*, and the fungal pathogen *Ustilago maydis* (Harbut et al. 2012; Allen et al. 2014; Chen et al. 2014; Pinter et al. 2019). In Arabidopsis, *SPP* was shown to be induced upon ER stress (Iwata et al. 2010). Thus, we tested *SPP* expression in *pi4kβ1,2*. As shown in Fig. 7a, about 2.5-fold upregulated expression of *SPP* was observed in *pi4kβ1,2*. Further, the *pi4kβ1,2* suppressors 79–1 and 171–1 showed complete suppression of *SPP* upregulation (Fig. 7a). However, 145–1, which is comparatively a weak suppressor of *pi4kβ1,2* (Fig. 1), displayed *SPP* expression similar to *pi4kβ1,2* (Fig. 7a). These results indicate that *SPP* is induced in *pi4kβ1,2* and that mutations in *SPP* are capable of suppressing this upregulated *SPP* expression seen in *pi4kβ1,2*.

### *pi4kβ1,2* displays heightened ER stress sensitivity and the spp mutations reduce the ER stress sensitivity of *pi4kβ1,2* and WT

The enhanced *SPP* expression prompted us to test whether the *spp* and *pi4kβ1,2* plants show altered ER stress response. When grown on ½ MS plates supplemented with the ER stress inducing chemical dithiothreitol (DTT), *pi4kβ1,2* exhibited enhanced sensitivity to DTT with seedlings appearing yellow and dying on plates supplemented with 2 mM DTT (Fig. 7b). In comparison, the suppressors 171–1 and 79–1 can survive better on plates supplemented with 2 mM DTT. 145–1 displayed marginal suppression of *pi4kβ1,2*’s DTT sensitivity (Fig. 7b).

We further tested whether the *spp* single mutants displayed reduced sensitivity to DTT. As shown in Fig. 7c-j, the *spp-3* and *spp-5* single mutant seedlings grew longer roots on DTT containing plates compared to WT. However, *spp-4* displayed a similar sensitivity to DTT as WT. Further, while expression of the ER-stress marker genes *Immunoglobulin*-*Binding Protein* (*BiP*) *1,2, BiP3* and *Endoplasmic Reticulum-localized DnaJ family 3B* (*ERdj3B*) were elevated in *pi4kβ1,2*, suppressors 79–1 and 171–1 displayed largely suppressed expression of these ER stress marker genes (Fig. 7k-m). 145–1 displayed a similar or mildly suppressed expression of these genes. These results uncover the antagonistic contributions of PI4Kβ and SPP in plant ER stress responses.

### *spp* single mutants have WT level of immunity

**Fig. 8:**
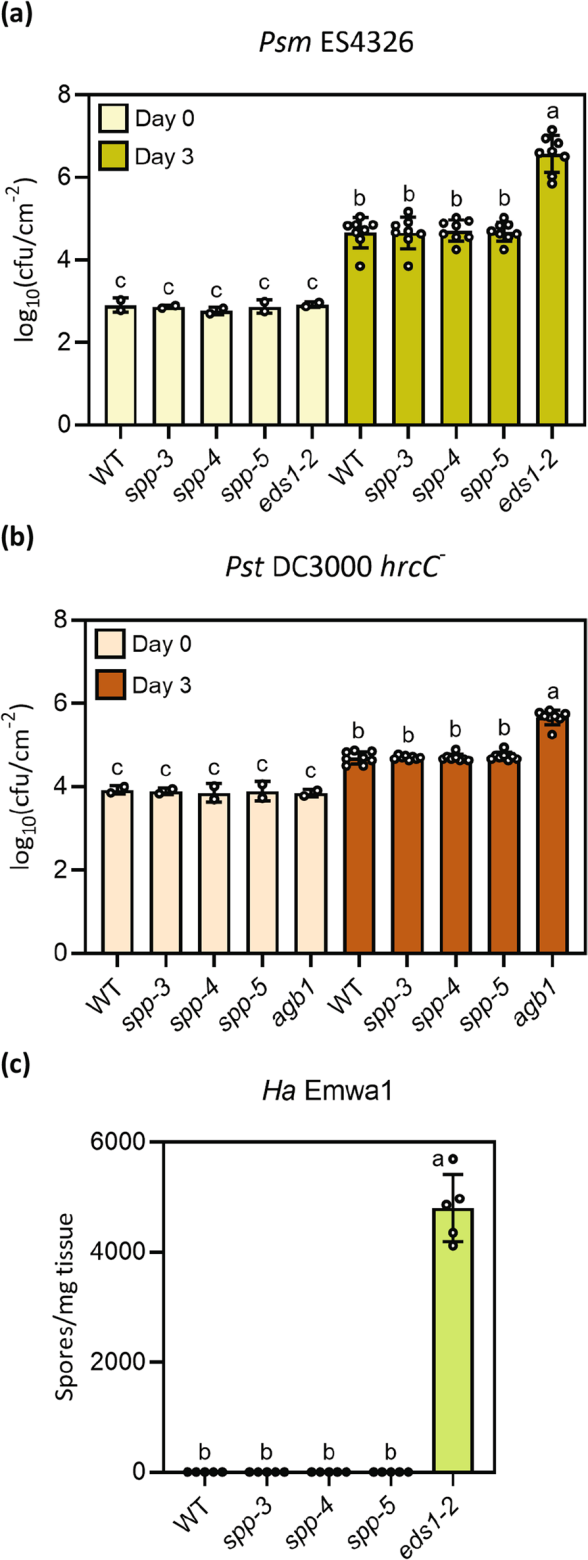
*SPP* single mutants display WT level of immunity against pathogens. **a** Quantification of *Psm* ES4326 growth in leaves of the indicated genotypes. Plants were infiltrated with bacterial suspension at OD_600_ = 0.0001, and bacterial titer was measured on day 0 and day 3 post infiltration. The error bars represent SD of the biological replicates (Day 0: *n* = 2; Day 3: *n* = 8). Genotypes denoted with the different letters have significant difference (*p* < 0.01) as determined using a one-way ANOVA with post-hoc Tukey’s Honestly Significant Difference test. **b** Quantification of *Pst* DC3000 *hrcC*^*−*^ growth in leaves of the indicated genotypes. Plants were infiltrated with bacterial suspension at OD_600_ = 0.001, and bacterial titer was measured on day 0 and day 3 post infiltration. The error bars represent SD of the biological replicates (Day 0: *n* = 2; Day 3: *n* = 8). Genotypes denoted with the different letters have significant difference (*p* < 0.05) as determined using a one-way ANOVA with post-hoc Tukey’s Honestly Significant Difference test. **c** Quantification of *Ha* Emwa1 growth on plants of the indicated genotypes. The error bars represent SD of the biological replicates (*n* = 5). Genotypes denoted with the different letters have significant difference (*p* < 0.01) as determined using a one-way ANOVA with post-hoc Tukey’s Honestly Significant Difference test

*SPP* was shown to be upregulated in seedlings treated with the immune related plant phytohormone N-hydroxypipecolic acid (NHP; Yildiz et al. 2021) or treatment with pathogen associated molecular patterns flg22 and elf18 (Bjornson et al. 2021) (Table 1). To examine the role of SPP in immunity, infection experiments were performed using the *spp* single mutants. The *spp* alleles displayed WT levels of immunity against the virulent bacterial pathogen *Pseudomonas syringae* pv. *maculicola* ES4326, the type III secretion system deficient *Pseudomonas syringae* DC3000 pv. *tomato hrcC*^*−*^ which triggers pattern triggered immunity (PTI), or the avirulent *Ha* Emwa1 which triggers RPP4 mediated effector triggered immunity (ETI) (Fig. 8). Thus, partial loss of SPP does not lead to observable defects in basal immunity, PTI or ETI.

**Table 1:** *SPP* is upregulated in response to treatment with NHP, flg22 and elf18

Elicitor (concentration)	Time point (hours)	*SPP* expression FC in WT (treatment vs. control)	Source
NHP (10 μM)	24	2.84	(Yildiz et al. 2021)
Flg22 (1 μM)	3	2.79	(Bjornson et al. 2021)
Elf18 (1 μM)	3	2.87	(Bjornson et al. 2021)

The table displays the *SPP* expression in fold change (FC) in response to treatment with the indicated elicitors. The data is sourced from Bjornson et al. 2021 and Yildiz et al. 2021

### Human *SPP* ortholog can complement the phenotypes of the suppressor 79–1

**Fig. 9:**
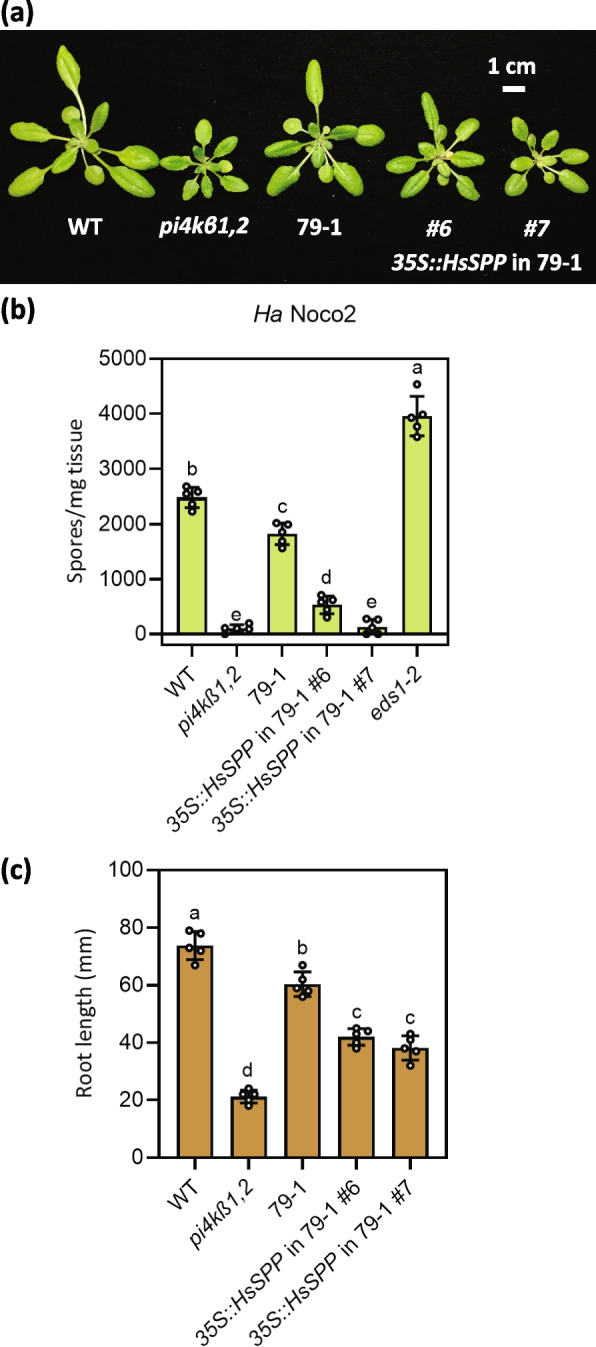
*HsSPP* can complement the phenotype of the suppressor 79–1. **a** Morphology of 4-week-old WT, *pi4kβ1,2*, 79–1, and two independent *35S::HsSPP* transgenic lines in the 79–1 background. **b** Quantification of *Ha* Noco2 growth on plants of the indicated genotypes. The error bars represent SD of the biological replicates (*n* = 5). **c** Root lengths of 10-day-old plate-grown seedlings of the indicated genotypes. The error bars represent SD of the replicates (*n* = 5). In (**b**-**c**) the letters indicate significant difference between the different genotypes as determined using a one-way ANOVA with post-hoc Tukey’s Honestly Significant Difference (HSD) test. Genotypes denoted with the different letters have significant difference (*p* < 0.05)

From the phylogenetic tree shown in Fig. 6b, Arabidopsis SPP is the most similar to the human SPP ortholog, HsSPP. In order to test whether HsSPP can perform the functions of Arabidopsis SPP, we performed a heterologous complementation test by transforming the 79–1 suppressor mutant with 35S promoter driven *HsSPP* cDNA clone (*35S::HsSPP*). Transgenic plants displayed partial reversion to *pi4kβ1,2*-like dwarf phenotype (Fig. 9a). Further, the transgenic plants displayed autoimmunity and root length defect close to the *pi4kβ1,2* mutant (Fig. 9b, c). These results suggest that human SPP is functionally similar to Arabidopsis SPP.

## Discussion

Signal Peptide Peptidase (SPP) and SPP-like (SPPL) proteins were first identified in humans (Weihofen et al. 2002). Since then, they have been found in animals including mouse (Urny et al. 2003), *Caenorhabditis elegans* (Grigorenko et al. 2004), Drosophila (Casso et al. 2005), and zebrafish (Krawitz et al. 2005). SPP was shown to be essential for development in *C. elegans*, Drosophila, zebrafish and mice since the knockout or knockdown mutants were not able to survive to maturity (Grigorenko et al. 2004; Casso et al. 2005; Krawitz et al. 2005; Aizawa et al. 2016). In parallel, Arabidopsis SPP and SPPLs were identified and SPP was shown to be an essential protein localized to the ER (Tamura et al. 2008; Han et al. 2009).

SPP’s initially characterised function was in the cleavage of signal peptides left over in the ER membrane after the action of signal peptidases (Weihofen et al. 2002). In addition, SPP also contributes to the clearing of misfolded membrane proteins (Schrul et al. 2010). Along with signal peptidases, SPP contributes to the sequential processing of type II membrane proteins with large luminal domains during intramembrane proteolysis (Mentrup et al. 2020, 2024). Further, type II tail-anchored membrane proteins with short luminal domains can be directly processed by SPP (Boname et al. 2014; Mentrup et al. 2017). Lastly, SPP was shown to be involved in ER-associated protein degradation (ERAD) for quality control of misfolded proteins that have accumulated in the ER (Loureiro et al. 2006; Lee et al. 2010; Chen et al. 2014).

The role of SPP and SPPLs have been extensively studied in animal systems. However, the functions of SPPs in plants are largely unclear. The Arabidopsis SPP was shown to be essential for the development of viable pollen (Han et al. 2009). Recently, it was shows that a nodule-specific SPP of *Medicago truncatula* is required for symbiosis with rhizobia (Yang et al. 2023). Here, we found that partial loss-of-function mutations in Arabidopsis *SPP* lead to suppression of *pi4kβ1,2* autoimmunity. In addition, these mutations can suppress its short root and root hair defects. This is noteworthy, since mutation in *EDS1* or a gene required for SA biosynthesis only partially suppresses *pi4kβ1,2*’s immunity and rosette size, but not its root defects (Šašek et al. 2014; Thulasi Devendrakumar et al. 2023). In a previous study, *SPP* was shown to be transcriptionally upregulated in Arabidopsis plants with induced ER stress (Iwata et al. 2010). Further, *SPP* was also induced in response to treatment with immune elicitors, indicating a potential role of SPP in mediating immune responses (Bjornson et al. 2021; Yildiz et al. 2021). In this study we found that *SPP* expression is upregulated in *pi4kβ1,*2. While *pi4kβ1,2* shows high sensitivity to ER stress and upregulation of ER stress marker genes, mutations in SPP can suppress *pi4kβ1,2*’s heightened ER stress in suppressors 79–1 and 171–1. The *spp-4* allele does not seem to suppress the ER stress responses likely because it is a weaker loss-of-function allele. While SPP was seen to be upregulated in response to treatment with immune elicitors, the *spp* single mutants did not display detectable enhanced susceptibility to pathogens. Thus, it is possible that elevated ER stress is the main defect of *pi4kβ1,*2, which can be alleviated with reduced SPP activity in both rosette and roots. Its autoimmunity could be downstream of such ER stress, explaining the partial reliance of the autoimmunity of *pi4kβ1,2* on SA or EDS1 and further explain the lack of an immune defect in the *spp* single mutants.

Further, it is interesting that mutations in *SPP* can lead to the formation of recessive partial loss of function alleles as found in suppressors 79–1 and 145–1, as well as a dominant-negative allele found in suppressor 171–1. All three suppressors carry *spp* alleles that result in single amino acid substitution mutations in proximity to the SPP transmembrane helices containing the active sites. However, these mutations result in varying degrees of suppression of *pi4kβ1,*2 phenotypes. All three suppressors show suppression of its autoimmunity and expression of the *PR1* and *PR2* immune marker genes. However, only suppressors 79–1 and 171–1 show suppression of the expression of *ICS1*, *EDS5*, ER stress marker genes, and the DTT induced ER stress sensitivity of *pi4kβ1,*2. Furthermore, only the *spp-3* and *spp-5* single mutants (spp alleles from suppressors 79–1 and 171–1 respectively) show reduced sensitivity to DTT induced ER stress. It is still unclear why these mutations result in SPP variants that are dominant-negative or recessive. Human SPP has been shown to function as a tetramer with their N-terminal regions being required for homo-oligomer formation with the catalytic C-termini present at the periphery of the SPP complex (Miyashita et al. 2011). SPP variants that disrupt the overall structure of the complex inhibit its catalytic activity, thereby having a dominant negative effect on SPP function (Miyashita et al. 2011). The dominant-negative allele identified in our screen could have similar mechanism. While these mutations could affect SPP’s function, it is also possible that these amino acid changes may negatively affect the stability of SPP. Further work to study the structure of the Arabidopsis SPP is required to understand the consequence of the amino acid changes on SPP structure and function.

From the phylogenetic tree shown in Fig. 6b, SPP is the most similar to the HsSPP. We further observed that HsSPP can complement the phenotype of suppressor 79–1. This suggests that HsSPP is able to target the Arabidopsis SPP substrate(s) required for *pi4kβ1,*2 phenotypes, supporting a highly conserved mechanism of SPP function across kingdoms.

We observed that SPP transiently expressed in *Nicotiana benthamiana* is localized to the ER, agreeing with a previous study (Tamura et al. 2008). However, in addition to the ER localization, we also observed puncta along the ER network. It is currently unclear what this punctate localization is and how it is relevant to SPP function. These puncta could either be membrane-bound organelles involved in the endomembrane system or nanodomains containing a high local concentration of SPP. Future co-localization experiments with other fluorescent markers can help distinguish between these possibilities and provide clues regarding the function of these puncta localized SPP. PI4Kβ1 was shown to be localized to distinct compartments of the trans Golgi network in root hair cells (Preuss et al. 2006; Kang et al. 2011). However, in these studies co-localization with marker constructs was lacking. Here we found that PI4Kβ1 transiently expressed in *N. benthamiana* is localised to the ER. This difference in localization could be attributed to the different tissues used in the localization studies. It is interesting that both SPP and PI4Kβ1 are ER localized. This shared localization may potentially allow for SPP to be activated by the loss of the PI4Kβs in *pi4kβ1,2*.

PI4Kβs are involved in the biosynthesis of phosphatidylinositol 4-phosphate (PI4P) (Preuss et al. 2006). Perturbed lipid homeostasis in the ER has been shown to result in the activation of the UPR either due to its effect on ER protein folding and transport or as a direct consequence of the altered lipid composition leading to lipid bilayer stress (Volmer & Ron 2015; Fun & Thibault 2020). Studies have also shown that ER stress, cell death, and plant immunity are intricately linked (Kørner et al. 2015; Ruberti et al. 2015; Manghwar & Li 2022). It is interesting that loss of the ER localized PI4Kβs trigger immune responses and ER stress that is dependent on another ER resident protein, SPP. It is possible that the perturbations in the levels of PI4P lead to heightened ER stress in *pi4kβ1,2* and SPP functions in this ER stress response. However, the exact molecular mechanism how this ER stress contributes to *pi4kβ1,2* autoimmunity remains unclear. Further work to identify the relevant substrates of SPP will help characterize its role in ER stress and immunity*.*

## Materials and methods

### Plant materials and growth conditions

All plants used in this study are in the *Arabidopsis thaliana* Col-0 ecotype. Col-0 is referred to as WT. The *pi4kβ1,2* mutant used in this study has been previously described (Preuss et al. 2006). Col-0 *eds1-2* (referred to as *eds1-2* in this study) and *agb1-2* (referred to as *agb1* in this study) that were used as susceptible controls in infection assays were previously described (Zhang et al. 2003; Ullah et al. 2003; Bartsch et al. 2006). All other lines were generated in this study. The *spp* single mutants were isolated by crossing the original suppressors 79–1, 145–1, and 171–1 to WT. From the segregating F2 populations the *spp* single mutants, *spp-3*, *spp-4*, and *spp-5*, that lacked the *pi4kβ1* and *pi4kβ2* T-DNA insertions were isolated. The primers used for genotyping are listed in Table S1. These *spp* single mutants were used for the root length based DTT sensitivity assays in Fig. 7, and for the infection assays in Fig. 8.

Seeds were soaked in 15% Chlorox® Original Concentrated Bleach supplemented with 0.1% Tween-20 for five minutes for surface sterilization. The seeds were then washed three times with sterile water. The seeds were then suspended in sterile 0.1% agar and stratified by storing them at 4 °C for at least two days.

To measure root length and for the root length based DTT sensitivity assays, standard square plates with ½ Murashige and Skoog (MS) media (pH 5.7) supplemented with 0.5% sucrose, 1% agar, and the appropriate concentration of sterile DTT were used. Sterilized seeds were exposed to light for 6 h and then sown on the plates. The plates were then placed vertically in a growth chamber with 12 h light and 12 h dark conditions. For DTT sensitivity assays based on seedling survival, plates were made with ½ MS media (pH 5.7) supplemented with 0.6% agar, 0.5% sucrose and the appropriate concentration of sterile DTT.

For sowing on soil, stratified seeds were exposed to light for 4 h or overnight. They were then planted on autoclaved Sunshine® Mix #4 aggregate. The flats were covered with a plastic dome for two to three days until the seeds germinated. The dome was then removed, and the plants were grown at 22 °C and long day conditions (16 h light and 8 h dark) for most purposes. For bacterial infection assays, seedlings were grown under long day conditions for two weeks, transplanted into single pots and grown for a further two weeks under short day conditions (8 h light and 16 h darkness) for two more weeks and then used. To grow plants for *Hyaloperonospora arabidopsidis* (*Ha*) Noco2 infection assays, the seedlings were grown for 12–14 days after sowing under long day conditions. The plants were then inoculated with the *Ha* Noco2 spore suspension, covered with a clear dome and then grown for an additional 7 days in a growth chamber set to 18 °C and 12 h light and 12 h darkness.

For all assays, seeds of all the genotypes to be compared were harvested from plants grown at the same time under identical conditions.

### EMS mutagenesis and screening M2 populations

Ethyl methanesulfonate (EMS) mutagenesis was carried out as previously described (Li & Zhang 2016). The suppressors 79–1, 145–1, and 171–1 were identified from vertical plate screen of the EMS mutagenized M2 population as described in Thulasi Devendrakumar et al. 2024.

### DNA isolation, next generation sequencing and data analysis

Total genomic DNA isolation for Next Generation Sequencing was performed as described in Thulasi Devendrakumar et al. 2024, 2023. The DNA was sequenced by Novogene using the Illumina® NovaSeq™ 6000 sequencing platform. The resulting NGS raw reads were analyzed using a variant discovery pipeline based on GATK Best Practices run on the Compute Canada Cedar cluster (Huang et al. 2019; van der Auwera & O’Connor 2020).

### Plasmid construction and primers

The *NP::SPP* construct was constructed by amplifying the *SPP* gene and its promoter (1012 bases upstream of *SPP*’s start codon) and cloning it into a promoterless binary vector. The *35S::SPP-mCitrine* construct was constructed by amplifying the *SPP* gene without the stop codon and cloning it into a 35S promoter driven binary vector with a C-terminal mCitrine tag. The *NP::HA-mNG-PI4Kβ1* construct was created by amplifying the *PI4Kβ1* gene and its promoter (1633 bases upstream of *PI4Kβ1*’s start codon) and cloning it into a binary vector upstream of the *HA-mNG*. Subsequently, *PI4Kβ1* gene was amplified and cloned into the vector downstream of the *HA-mNG*. For these three constructs, Arabidopsis WT DNA was used as template and the amplicons were amplified using NEB Q5® High-Fidelity DNA polymerase. The *35S::HsSPP* was cloned by amplifying the *HsSPP* CDS (Isoform 1) and cloning it into a 35S promoter driven binary vector. RNA was extracted from Caco-2/TC-7 human colon adenocarcinoma cell line. The cDNA generated from this RNA was used as the template to amplify *HsSPP* CDS. The primers used for cloning are listed in Table S1.

### Infection assays

For oomycete *Ha* Noco2 and *Ha* Emwa1 infection assays, seedlings were sprayed with the spore suspension in water (50,000 spores/ml). 7 days after inoculation, whole above ground seedling tissue was harvested and vortexed with sterile water. The suspended *Ha* Noco2 spores were then counted using a hemocytometer. Bacterial infection assays were performed as described in Thulasi Devendrakumar et al. 2023. In brief, *Pseudomonas syringae* pv. *maculicola* ES4326 and *Pseudomonas syringae* pv. *tomato* DC3000 *hrcC*^*−*^ bacterial suspensions were syringe infiltrated into the leaves. Leaf disks were collected on Day 0 (day of infiltration) and Day 3 (three days after infiltration), ground in 10 mM MgCl_2_, serially diluted and plated on LB plates to measure the colony forming units (cfu) per cm^2^ of the leaf.

### Root length measurement

For regular root length measurement 10-day old seedlings grown on vertical ½ MS plates were used. The seedlings were removed from the agar surface, the root was laid straight and measured manually using a ruler. The distance between the base of the hypocotyl to the root tip was recorded as the root length. For DTT sensitivity assays, the root lengths of 14-day old seedlings were measured.

### Quantitative RT-PCR

30–40 mg of leaves were collected from 4-week-old soil grown plants in 2 ml tubes containing 2 glass beads and frozen in liquid nitrogen. The tissue was then ground to a fine powder using a Precellys tissue homogenizer. The total RNA was extracted using the EZ-10 Spin Column kit (BioBasic, Markham, Canada). 1 μg of RNA was then reverse transcribed into cDNA using the ABM EasyScript™ cDNA Synthesis Kit following the manufacturer’s protocol and the cDNA was used for RT-qPCR. TB Green® Premix Ex Taq™ II mastermix was used for the RT-qPCR. BioRad’s CFX Connect™ Real-Time PCR Detection System was used for the qPCR run. The primers used for the reverse transcription and qPCR are listed in Table S1.

### Confocal laser scanning microscopy

To detect fluorescence of mCitrine, mNeonGreen (mNG) or Cyan Fluorescent Protein (CFP) fusion proteins confocal laser scanning microscopy was performed using a TCS SP8 Falcon system, with 63 × water immersion objective and LASX 3.5 software (Leica Microsystems, Wetzlar, Germany). The endoplasmic reticulum (ER) marker ER-ck (CFP; Nelson et al. 2007) was used for co-localization. For excitation of mCitrine, mNG, and CFP, a pulsed white light laser was used with laser lines set to 488 nm, 514 nm or 458 nm, respectively. Fluorescence emission was detected using HyD SMD detectors with detection windows ranging from 525 to 560 nm for mCitrine, 500 to 540 nm for mNG, and 465 to 485 nm for CFP. Images were recorded in sequential scanning mode to avoid bleach through of CFP signals into the mCitrine channel.

### Phylogenetic analysis and tree construction

The amino acid sequences of the *Arabidopsis thaliana* (6 proteins) and *Homo sapiens* (human; 5 proteins) SPP and SPPLs were obtained from TAIR (Arabidopsis) and NCBI (Human). The full-length sequences of the proteins encoded by the most abundant splice variant of each of the genes were aligned using Muscle align in MEGA X. This alignment was then used to predict the evolutionary history of the SPP and SPPLs.

The evolutionary history was inferred using the Maximum Likelihood method and JTT matrix-based model (Jones et al. 1992). The tree with the highest log likelihood (-11827.01) is shown. The percentage of trees in which the associated taxa clustered together is shown next to the branches (1000 bootstrap replicates). Initial tree(s) for the heuristic search were obtained automatically by applying Neighbor-Join and BioNJ algorithms to a matrix of pairwise distances estimated using the JTT model, and then selecting the topology with superior log likelihood value. The tree is drawn to scale, with branch lengths measured in the number of substitutions per site. There were 844 positions in total in the final dataset. Evolutionary analyses were conducted in MEGA X (Kumar et al. 2018).

### Supplementary Information

**Additional file 1: Fig. S1.** *NP::SPP* can complement the phenotype of the suppressor 171-1. (a) Morphology of 4-week-old WT, *pi4kβ1,2*, 171-1, and two independent *NP::SPP* transgenic lines in the 171-1 background. (b) Quantification of *Ha* Noco2 growth on plants of the indicated genotypes. The error bars represent SD of the biological replicates (*n*=5). (c) Root lengths of 10-day-old plate-grown seedlings of the indicated genotypes. The error bars represent SD of the replicates (*n*=5). In Fig. S1b,c, the letters indicate significant difference between the different genotypes as determined using a one-way ANOVA with *post hoc* Tukey’s Honestly Significant Difference (HSD) test. Genotypes denoted with the different letters have significant difference (*p*<0.05).

**Additional file 2.**

## Data Availability

For Agrobacterium strains, plasmids, or Arabidopsis genotypes generated in this study, please contact the corresponding author who will make it available upon reasonable request.

## Abbreviations

AGB1: Arabidopsis GTP binding protein Beta 1
cDNA: Complemetary DNA
CFP: Cyan fluorescent protein
cfu: Colony forming units
DNA: Deoxyribonucleic acid
DTT: Dithiothreitol
EDS1: Enhanced Disease Susceptibility 1
EDS5: Enhanced Disease Susceptibility 5
EMS: Ethyl methanesulfonate
ER: Endoplasmic reticulum
ERAD: Endoplasmic reticulum–associated protein degradation
ETI: Effector Triggered Immunity
GATK: Genome Analysis Toolkit
Ha: *Hyaloperonospora arabidopsidis*
Hs: Homo sapiens
ICS1: Isochorismate Synthase 1
mNG: mNeonGreen
MS: Murashige-Skoog
NGS: Next Generation Sequencing
NP: Native promoter
PI4K: Phosphatidylinositol 4-kinase
PI4P: Phosphatidylinositol 4-phosphate
PR1: Pathogenesis-Related gene 1
PR2: Pathogenesis-Related gene 2
*Psm*: *Pseudomonas syringae* pv. *maculicola*
*Pst*: *Pseudomonas syringae *pv. *tomato*
PTI: Pattern-Triggered-Immunity
RT-qPCR: Reverse transcription- quantitative real time polymerase chain reaction
SA: Salicylic acid
SPP: Signal Peptide Peptidase
SPPL: SPP-Like
TL: Transmitted Light
UPR: Unfolded Protein Response
WT: Wild-type
