# Supplementary material for: Signal Peptide Peptidase and PI4Kβ1/2 play opposite roles in plant ER stress response and immunity
Source: Stress Biol. 2024 Mar 20;4(1):20. doi: 10.1007/s44154-024-00155-z (PMC10954597; doi:10.1007/s44154-024-00155-z)
Supplement: Supplementary file 1 — Additional file 1: Fig. S1. NP::SPP can complement the phenotype of the suppressor 171-1. (a) Morphology of 4-week-old WT, pi4kβ1,2, 171-1, and two independent NP::SPP transgenic lines in the 171-1 background. (b) Quantification of Ha Noco2 growth on plants of the indicated genotypes. The error bars represent SD of the biological replicates (n=5). (c) Root lengths of 10-day-old plate-grown seedlings of the indicated genotypes. The error bars represent SD of the replicates (n=5). In Fig. S1b,c, the letters indicate significant difference between the different genotypes as determined using a one-way ANOVA with post hoc Tukey’s Honestly Significant Difference (HSD) test. Genotypes denoted with the different letters have significant difference (p<0.05). [file 44154_2024_155_MOESM1_ESM.pdf]

Figure S1

(a)

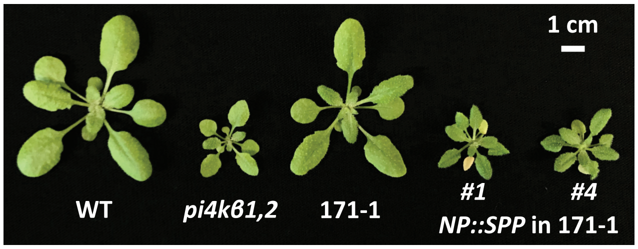

(b)

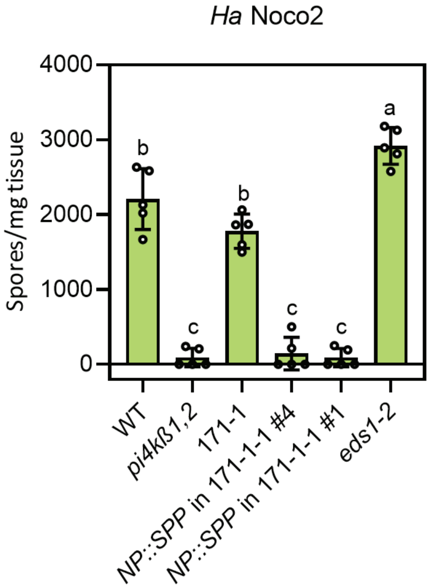

(d)

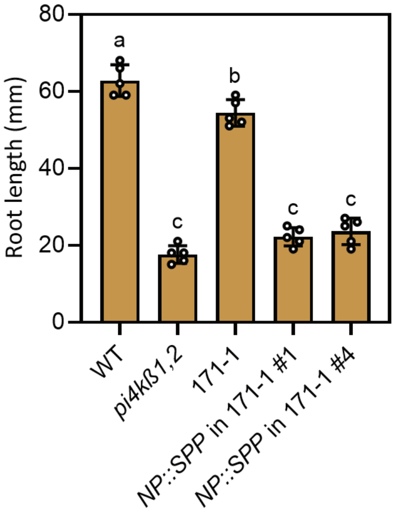

Fig. S1 *NP::SPP* can complement the phenotype of the suppressor 171-1.

(a) Morphology of 4-week-old WT, *pi4kβ1,2*, 171-1, and two independent *NP::SPP* transgenic lines in the 171-1 background.

(b) Quantification of *Ha* Noco2 growth on plants of the indicated genotypes. The error bars represent SD of the biological replicates (n=5).

(c) Root lengths of 10-day-old plate-grown seedlings of the indicated genotypes. The error bars represent SD of the replicates (n=5). In Fig. S1b,c, the letters indicate significant difference between the different genotypes as determined using a one-way ANOVA with *post hoc* Tukey's Honestly Significant Difference (HSD) test. Genotypes denoted with the different letters have significant difference ($p<0.05$).
